# Unsupervised Mitral Valve Tracking for Disease Detection in Echocardiogram Videos

**DOI:** 10.3390/jimaging6090093

**Published:** 2020-09-09

**Authors:** Kazi Tanzeem Shahid, Ioannis Schizas

**Affiliations:** Department of Electrical Engineering, University of Texas at Arlington, Arlington, TX 76019, USA

**Keywords:** ultrasound, echocardiography, mitral valve, apical 4 chamber, unsupervised

## Abstract

In this work, a novel algorithmic scheme is developed that processes echocardiogram videos, and tracks the movement of the mitral valve leaflets, and thereby estimates whether the movement is symptomatic of a healthy or diseased heart. This algorithm uses automatic Otsu’s thresholding to find a closed boundary around the left atrium, with the basic presumption that it is situated in the bottom right corner of the apical 4 chamber view. A centroid is calculated, and protruding prongs are taken within a 40-degree cone above the centroid, where the mitral valve is located. Binary images are obtained from the videos where the mitral valve leaflets have different pixel values than the cavity of the left atrium. Thus, the points where the prongs touch the valve will show where the mitral valve leaflets are located. The standard deviation of these points is used to calculate closeness of the leaflets. The estimation of the valve movement across subsequent frames is used to determine if the movement is regular, or affected by heart disease. Tests conducted with numerous videos containing both healthy and diseased hearts attest to our method’s efficacy, with a key novelty in being fully unsupervised and computationally efficient.

## 1. Introduction

Echocardiograms refer to ultrasound images of the heart. As the heart is one of the most vital organs in the human body, it is crucial to ensure its health and by monitoring it regularly. Out of the various imaging technologies available in this regard, ultrasound is very popular for its simplicity, noninvasive nature, its lack of harmful effects, and its ability to detect a wide range of common heart diseases. The blood circulates in two major loops: pulmonary and systemic circulation. Deoxygenated blood from the body is delivered to the right atrium by two primary veins. Afterwards, through the right atrioventricular (or tricuspid) valve, it is pumped to the right ventricle and pulmonary arteries in the lungs. The now-oxygenated blood returns from the lungs through the pulmonary veins to the left atrium. Once there, it is pumped thorough the left atrioventricular (or mitral) valve to the left ventricle, and further to the aorta to supply the whole body. During the diastolic phase the left ventricle musculature relaxes, and the inner space of the chamber expands, which allows blood from the left atrium to enter the left ventricle [1]. As the ventricle fills up with blood, the second phase begins (systole), during which the left ventricle contracts and ejects the blood to the aorta. The same cycle is observed in the right cavities of the heart; however, the left ventricle generates considerably higher pressures and its walls are thicker than these of the right ventricle. One reason for this, is that the left ventricle pumps blood throughout the body, and thus requires higher muscle definition, as opposed to the right ventricle pumping the blood only to the lungs. Both the mitral and tricuspid valve prevent backward blood flow during the systole phase (from the ventricles to the atria). Even so, due to higher pressures present in the left ventricle, the mitral valve function is more crucial than the tricuspid valve, thus any disease affecting its function needs to be addressed to ensure a person’s health [2,3,4].

Some very common heart diseases affecting the mitral valve include mitral stenosis, where the mitral valve is narrowed mainly due to mitral valve annulus calcification, or deposits of calcium on the mitral valve leaflets. Another disease is endocarditis, which causes bacteria to grow around the leaflets, leading to their degeneration. These changes impede sufficient blood flow through the valve, and force inadequate quantities of blood to be pumped into the left ventricle, which also causes blood retention in the pulmonary vasculature [5].

Another common affliction is mitral regurgitation, where the mitral valve is unable to close fully during the systolic phase. This causes some part of the blood being pumped by the left ventricle to the aorta, to instead divert back to the left atrium. This will ultimately also cause the body to receive insufficient blood, and can also cause left ventricular overload.

As all these cases affect movement of the mitral valve leaflets, the ability to track their movements is beneficial towards detecting the presence of any potential diseases that may affect the heart. Of the various angles through which the valve is observed in echocardiography, a well-known one is the apical 4 chamber view, where all four main chambers of the heart are visible. This allows a broad overview of the heart, as well as the ability to clearly see the movement of the left atrium, ventricle and mitral valve [6]. Furthermore, as the entire heart is in the frame, any involuntary motion will be comparatively less pronounced than in any other angle, as all other angles (arguably except for the apical 2 chamber view) will usually focus on a smaller part of the heart, thereby making smaller movements much more pronounced, and consequently disruptive.

There has been some progress made regarding tracking the mitral valve in echocardiography images. The work in [7] uses a curve fitting method to estimate the mitral valve and the left ventricular wall through image segmentation. Machine learning has been used effectively in fetal echocardiograms [8], where convolutional neural networks are used to apply image segmentation for tracking the mitral valve. Image segmentation in deep learning is also a researched topic [9], as well as having shallower neural networks for observing the mitral valve [10]. These schemes will usually take each frame of a video as a sample, while in our scheme for disease detection, each video is treated as a sample. This will cause our sample size to be much smaller, and it would thus be incompatible with neural networks which usually rely on very large datasets of samples. Another approach is to remove the background elements using an Alternating Direction Method of Multipliers (ADMM) to isolate only the relevant foreground [11], but it relies on prior dictionaries of features for both the foreground and background, which would prevent the scheme from being unsupervised.

Active contours [12] have been used for tracking the mitral valve [13,14]. One notable method [15] emulates the M-mode view to track movement across time, across a line that goes through the mitral valve leaflets. The M-mode is a view where a line is taken through two predetermined points, and any movement across the line will only be tracked. This would cause issues if the mitral valve movement is significant to the point of it moving beyond the given line.

Unsupervised mitral valve tracking has been studied, albeit on a smaller degree. Ref.  [16] tracks the mitral valve leaflet in an unsupervised manner, but must assume that the movement is restricted within a relatively small region, which might not necessarily be the case. 3D and 4D ultrasound has also been researched upon for segmentation of the mitral valve [17,18,19], offering the ability to create more accurate models for heart functionality and allowing clearer views of the mitral valve. However, the complexity of the equipment required to capture these images make its application more limited than 2D echocardiograms.

The motivation of our work is to not only create a highly robust scheme that detects the presence of mitral valve disease in a fully unsupervised manner, but to do so in a setting where the data has as little preprocessing as possible. We have acquired a highly challenging and diverse dataset, containing videos that were never acquired specifically for the purposes of our scheme. Furthermore, with requiring little to no preprocessing on the data, this scheme can be easily applied to any patient echocardiogram by someone with no expertise in our work. In situations where a seasoned cardiologist might not be present, a novice technician can apply this to acquired tests and obtain meaningful results, which can be helpful in, for example, rural areas where an expert is not readily available. It should be pointed out that even though color doppler [20], and 3D echocardiography exists that can possibly address this problem more efficiently, 2D echocardiography without Color Doppler is still widely used as it is cheaper, and it is also far simpler due to its lack of necessary specialized training compared to 3D echocardiography, especially TEE echocardiography which is also invasive. Thus, grayscale 2D echocardiograms are the data we have chosen to use for our scheme. Lastly, as mentioned previously, Ref. [16] can be considered unsupervised, but as it requires manual cropping of the data to a much smaller area around which the mitral valve resides, it is still strictly speaking a supervised technique. Our scheme, on the other hand, does not require this.

The structure of the paper follows next. Section 2.1 describes how the frames in an ultrasound video are binarized by Otsu’s thresholding, and two frames are chosen and combined to create a closed boundary around the left atrium. Section 2.2 discusses how the contour around the atrium is taken and used to find the centroid of the atrium. Section 2.3 illustrates the process behind taking protruding prongs from the centroid to estimate the locations of the mitral valve leaflets on a frame-by-frame basis. Section 2.4 shows how the position and movement of the mitral valve leaflets are calculated via an automatically determined variance threshold created by measuring the position of the mitral valve leaflets. Section 2.5 describes how the values obtained previously are used to detect the presence of disease of the heart monitored in the ultrasound video.

## 2. Proposed Method

### 2.1. Otsu’s Thresholding

Consider an ultrasound video with *N* frames, where each frame $F_{n}$, ∀n=1,…,N is an $R^{w\times h}$ array, where *w* and *h* represent horizontal pixel length (width) and vertical pixel length (height) respectively. The values in this array represent pixel values, where higher values correspond to whiter pixels. Otsu’s thresholding [21] is a method to choose a threshold which would turn the frame into a binary image, where 0’s and 1’s would correspond to pixel values below and above the threshold respectively. The threshold would be chosen in such a way that minimizes *intra-class* variance, meaning that the threshold would ensure that pixel values below and above the threshold would have minimal variance among themselves. If we wish to binarize an image, some parts of the image will be part of the foreground, and the rest will be in the background.

Consider the pixel values in a frame $F_{n}$ to be $p_{i}$, ∀i=1,…,w×h. For some threshold *t*, all pixel values below it will be considered part of the background $p_{b^{j}}$, $\forall j=1,\ldots ,P_{b}$ and all others will be part of the foreground $p_{f^{k}}$, $\forall k=1,\ldots ,P_{f}$. $P_{b}$ and $P_{f}$ represent the number of pixels in the background and foreground respectively, meaning that $P_{b}+P_{f}=w\times h$. Thus, for a given threshold *t*,

(1) $$\begin{array}{cc} p_{i}=p_{b^{j}}, & \;\mathrm{if}\;p_{i}<t\\ p_{i}=p_{f^{k}}, & \;\mathrm{if}\;p_{i}\geq t \end{array}$$

The value of the threshold *t*, is the one that minimizes the intra-class variance, which is a weighted sum of the variances of the foreground $\sigma_{f}^{2}$ and background $\sigma_{b}^{2}$. Specifically:   

(2) $$\begin{array}{cc} t & =\arg\min_{t}\left[w_{f}\left(t\right)\sigma_{f}^{2}\left(t\right)+w_{b}\left(t\right)\sigma_{b}^{2}\left(t\right)\right]\\ \mathrm{where},\; & w_{b}\left(t\right)=\frac{P_{b}}{P_{f}+P_{b}}\\ \; & w_{f}\left(t\right)=\frac{P_{f}}{P_{f}+P_{b}}\\ \; & \sigma_{b}^{2}\left(t\right)=\frac{\sum_{j=1}^{P_{b}}{\left(p_{b^{j}}-\mu_{b}\right)}^{2}}{P_{b}}\;\mathrm{where},\;\mu_{b}=\frac{1}{P_{b}}\sum_{j=1}^{P_{b}}p_{b^{j}}\\ \; & \sigma_{f}^{2}\left(t\right)=\frac{\sum_{j=1}^{P_{f}}{\left(p_{f^{k}}-\mu_{f}\right)}^{2}}{P_{f}}\;\mathrm{where},\;\mu_{f}=\frac{1}{P_{f}}\sum_{k=1}^{P_{f}}p_{f^{k}} \end{array}$$

Please note that the value of *t* affects the values of $P_{f}$ and $P_{b}$ via Equation (1). This effectively reduces the background noise present in the frame, while also binarizing the image and make its processing much faster.

After all the *N* frames are binarized, we obtain *N* binary arrays where each array has 1’s in the pixels where tissues are likely to be present. We thus obtain *N* binary frames $B_{n}$, ∀n=1,…,N. Note that in Equation (1), the frames were vectorized as part of the generalized formula. However, it is not necessary in the algorithmic implementation.

Separately, we apply background subtraction to $F_{n}$. Assume the background to be S, where each pixel of S is the median value across frames 1 through *N*, so $S=median(F_{1},F_{2},\cdots ,F_{N})$ on an entry-by-entry basis, ∀n=1,…,N. We thus obtain the background subtracted images $F_{S^{n}}=F_{n}-S$. We binarize these images using the same process in Equations (1) and (2) and obtain the binarized versions of $F_{S^{n}}$, denoting them as $B_{S^{n}}$.

**Figure 1:**
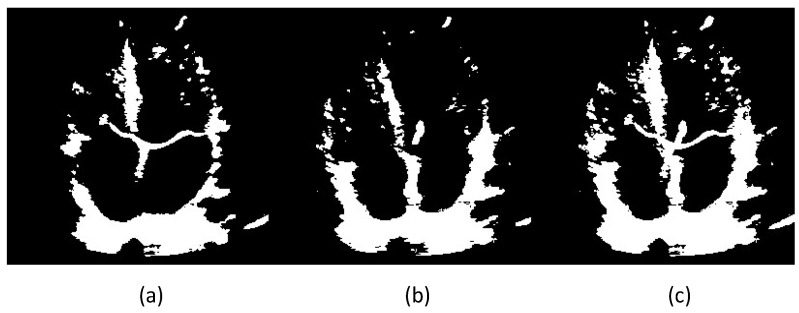
Two frames of a heart’s ultrasound video after binarizing, (**a**,**b**) are the two frames whose centroids are farthest from each other, (**c**) is obtained by combining the two, i.e., $B_{x}+B_{y}$.

After the previous step, we take the coordinates of the remaining pixels containing 1’s and find their centroid, and measure the Euclidean distance of the centroids across different frames. This is done so that we find the two frames that exhibit the maximum range of motion shown by the mitral valve, as the two frames with the farthest centroids will correspond to the frames where the mitral valve leaflets are fully open and closed. We assume the two frames to be $B_{S^{x}}$ and $B_{S^{y}}$. After finding these two frames, they are combined, along with the previously subtracted background. The final combined image is essentially $B_{x}+B_{y}$. We label this combined image as $C\in R^{w\times h}$. We consider the background subtracted version of this to be $C_{S}$. Figure 1 shows three images, where (a), (b) and (c) would be $B_{x}$, $B_{y}$ and C respectively. Please note that C allows us to observe a closed boundary around the left atrium, while even (a) is unable to do so, since the involuntary movement of the heart makes the interatrial septum to be out of the viewing plane. Reiterating, $B_{x}$ is the binarized version of $F_{x}$, and $B_{S^{x}}$ is the binarized version of $F_{S^{x}}$. The presence of S signifies the presence of background subtraction.

### 2.2. Contour and Atrial Centroid Estimation

Next, we design an unsupervised approach which determines the left atrium’s boundary automatically without human intervention. The muscles and other tissues making up the boundary of the left atrium might not be clearly defined in every frame. This is particularly problematic with the thinness of the interatrial septum, the wall between the two atria, which can be normally as thin as ≤1 mm. Because of this, a recursive loop is established to find a closed contour surrounding the left atrium.

We take the combined image $F_{x}+F_{y}$ and perform Otsu’s thresholding on it, with and without background subtraction. This is because in usual cases, the mitral valve is thinner compared to the muscles surrounding the left atrium, necessitating a lower threshold for the background subtracted image. Thus, we would obtain two thresholds, for the image with background ($F_{x}+F_{y}$) and without background ($F_{S^{x}}+F_{S^{y}}$), namely $l_{1}$ and $l_{2}$ respectively. Then, with the simple rationale that the left atrium will be situated at the bottom right corner in the apical 4 chamber view, we observe how many closed boundaries exist inside the bottom right corner of the image. As no chambers exist besides the left atrium in the image’s bottom right corner, any closed boundaries besides it will be formed by small noise artifacts. Thus, if a closed boundary exists that is adequately large, it would be sufficient to consider that as the left atrium. For our experiments, we consider a boundary that contains pixels more than 10% (parameter *p*) of the box’s length and height to be adequately large. This particular value was chosen on a trial-and-error basis(see details in Section 3.1.5). Let $b_{x}$ and $b_{y}$ denote the pixel lengths of the bottom right corner of the image, then $b_{x}=0.5\times w$ and $b_{y}=0.5\times h$.

If, however, we are unable to find a closed boundary that is large enough, it is safe to presume that the Otsu’s thresholds obtained were too large to create the boundary around the atrium. So, we implement a recursive loop, where the values of $l_{1}$ and $l_{2}$ are incrementally decreased by a small step size *c* until a closed boundary is obtained. Please note that with the lowering of the threshold, more and more noise will be added to the image, but they will generally be small specks, creating closed boundaries far smaller than the given 10% minimum. When a threshold value is obtained that creates the desired boundary, the centroid is calculated from it. Algorithm 1 delineates the recursive loop algorithm at work. The method used for finding closed boundaries was a combination of the Moore-Tracing algorithm, with Jacob’s stopping criterion [22].

It can be seen that the closed boundary is obtained once four conditions are met. The first two are to ensure that the closed boundary is large enough, the latter two ensure that the boundary is within the box. It should be noted also that in our experiments, it was observed that due to involuntary movement of the heart, there is a possibility the atrium may move beyond this box. Thus, for added safety, the box is taken to be slightly larger, taking 60% of the pixels on each dimension instead of 50%. Also, we set p=0.1 and c=0.005 in our experiments.

**Figure 2:**
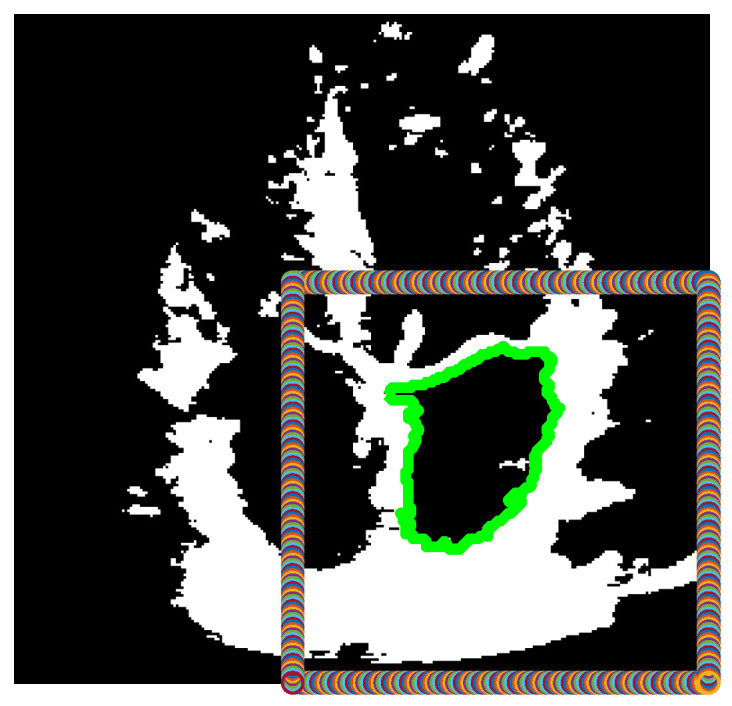
Binarized version of the combined frame, after finding a large contour (green) within the box defined in the bottom right corner of the image.

Figure 2 depicts the given algorithm finding the correct closed boundary around the left atrium, which is within the automatically generated box in the bottom right corner, where the left atrium always resides.
**Algorithm 1:** Recursive Estimation of the Closed Boundary of the Left Atrium1:Initialize values $l_{1}$, $l_{2}$ from binarizing matrices $F_{x}+F_{y}$ and $F_{S^{x}}+F_{S^{y}}$. Set step size *c*, percentage threshold *p*. Set box to be bottom right corner of image, input box length $b_{x}$ and height $b_{y}$. Coordinate of top left corner of box is {$c_{x}$,$c_{y}$}.2:**for**$s_{1}=0,1,\cdots$**do**3:  Set $l_{1}=l_{1}-c$4:  **for** $s_{1}=0,1,\cdots$ **do**
5:    Set $l_{2}=l_{2}-c$6:    Use updated $l_{1}$ and $l_{2}$ to obtain new $C:=B_{x}+B_{y}$ and $C_{S}:=B_{S^{x}}+B_{S^{y}}$.7:    Calculate number of closed boundaries $N_{B}$ in $C+C_{S}$.8:    **for** $i=1,\cdots ,N_{B}$ **do**9:      Set maximum and minimum coordinates along both axes to be $x_{max}$, $y_{max}$, $x_{min}$ and $y_{min}$ respectively.10:      If $x_{max}-x_{min}\geq p\times b_{x}$, $y_{max}-y_{min}\geq p\times b_{y}$, $x_{min}\geq c_{x}$ and $y_{max}\leq c_{y}$ end update. Otherwise continue.11:    **end for**12:  **end for**13:  If condition in Step 6 is still not met, reinitialize $l_{2}$.14:**end for**15:Calculate centroid of obtained closed boundary.

### 2.3. Creating Prongs

**Figure 3:**
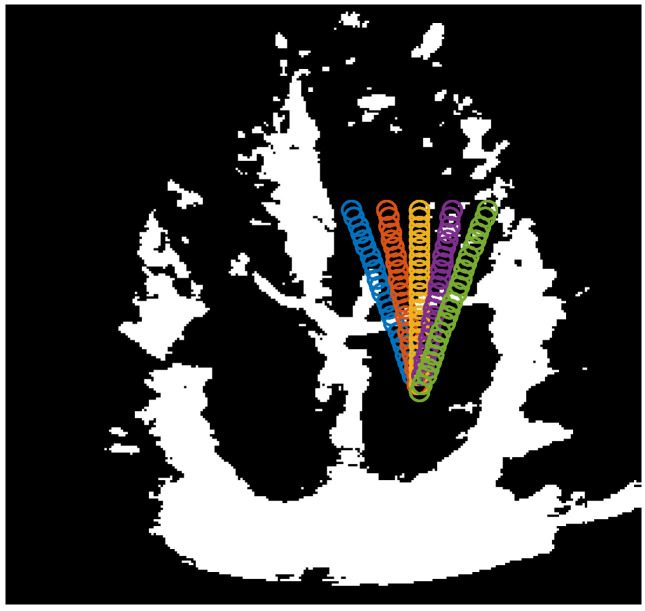
5 prongs centered at the atrium boundary centroid and spanning 40${}^{\circ }$.

After finding the centroid of the closed boundary, in the apical 4 chamber view, the mitral valve leaflets will always be above the centroid. Now that the left atrium’s location is known, we can safely presume that taking a group of prongs protruding from the centroid in the upward direction will meet the mitral valve leaflets. These prongs should form a cone within the width of the mitral valve. The length of these prongs should be long enough to ensure that even if the shrinking of the left ventricle during the systolic phase causes the mitral valve to move farther away from the atrium’s centroid, it will still be long enough to meet the valves. The advantage to keep this range as short as possible is that longer prongs need to be created using a higher number of points, affecting processing speeds. To this effect, the length of the prongs, as well as the density of points defining these prongs will depend on the size of the estimated contour of the left atrium. We have defined the length of the prongs as a percentage of the height of the contour around the left atrium, in other words, $y_{max}-y_{min}$ once the algorithm is complete. Thus, the value $l_{P}=25$, means 25% of $y_{max}-y_{min}$. The cone is set to span 40${}^{\circ }$ above the centroid. Figure 3 shows the prongs being generated. We have chosen the number of prongs to be 5.

### 2.4. Estimating Movement of Mitral Valve

The penultimate section of the proposed method is to estimate whether the mitral valve is open or not. If the mitral valve is closed, then the prongs protruding from the centroid of the left atrium will not only touch the mitral valve leaflets, but since the leaflets will be more or less horizontal, the points where the prongs will meet the mitral valve leaflets would be close to each other. This means that their coordinates will have little standard deviation σ among each other. Alternatively, if the mitral valve is open, then the prongs would be touching the valve leaflets at points more distant from each other, or touching nothing at all. This will cause the points to have a much higher σ among them. Therefore, if a threshold is determined where the value of σ of the points in a particular frame is less than that threshold, we can label the frame’s mitral valve to be ‘closed’, and the frame with a σ value above the threshold to be ‘open’. As the threshold can depend on key factors like the size of the left atrium, the amount of involuntary movement in the heart, the size of the opening of the mitral valve during the diastole phase, the angle of the viewing plane due to placement of the probe, this threshold can vary greatly depending on the patient. Thus, we developed a method to determine the threshold in an unsupervised manner.

Depending on the echocardiogram video, no matter what the values of the σ might be, it will always be the case that closed valves will have a much smaller value compared to valves that are open. So, if the values of σ across various frames are sorted in ascending order, the threshold is chosen to be the value just before the largest shift in the value of σ.

**Figure 4:**
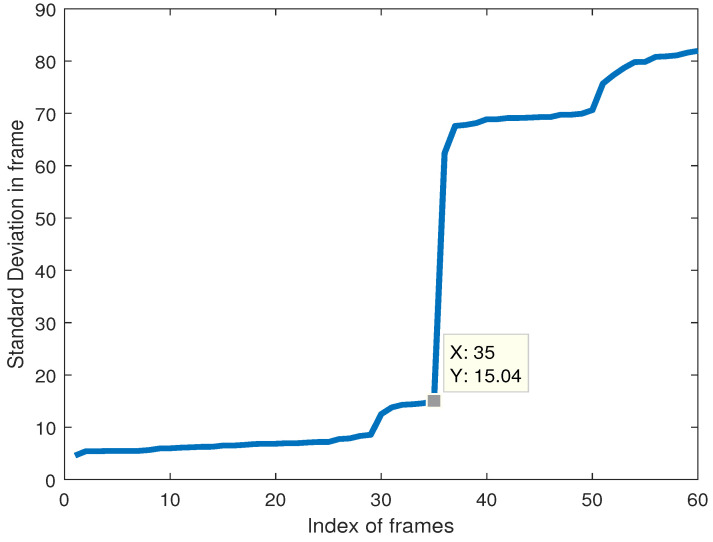
Coordinate standard deviation in a configuration involving 5 prongs within a 40-degree cone from the centroid.

Figure 4 shows the obtained values of σ for the video whose frames were used in Figure 1, Figure 2 and Figure 3. As we can see, the value of σ just before the largest shift across the frames is 15.04 in the 35th frame, so anything equal to or below this threshold will signify a ‘closed’ valve, and anything above it will be ‘open’.

### 2.5. Disease Estimation

After estimating the state of the mitral valve as open or closed, we use it to estimate whether the heart observed is healthy or diseased. We would consider a healthy heart to have an adequate number of frames with ‘open’ and ‘closed’ states, as well as an adequate number of consecutive frames with one state at a time. There are three possible scenarios being considered for the heart’s state:

(1) Too few consecutive frames switch between ‘open’ and ‘closed’ states multiple times. This would imply diseases like supraventricular tachycardia, atrial fibrillation, atrial flutter and other forms of arrhythmias [5].

(2) Too many frames in the ‘closed’ state. This would indicate diseases where the mitral valve leaflets have some form of blockage preventing the valve to open properly, such as mitral stenosis [23].

(3) Too many frames in the ‘open’ state. This would point to diseases where the mitral valve leaflets lack the ability to close fully during the systole phase, such as mitral prolapse [24].

For these effects, three thresholds are put in place. The first threshold measures how often states change twice in 3 consecutive frames. Instead of giving a fixed number for this, we set it as 5% of the number of frames of a given video, as the videos used have significantly different numbers of frames. The second and third threshold is set as 0.85, meaning that if more than 85% of the frames are either ‘open’ or ‘closed’, the heart will be classified as diseased. These thresholds are obtained on a trial-and-error basis, as will be explained in the following section.

## 3. Numerical Tests

For our numerical tests, 62 samples were obtained from 59 patients. The videos were obtained online from YouTube, as well as some taken from tests conducted under a local cardiology clinic named Cardiology Partners in Mansfield, Texas. The videos had 42 healthy and 20 diseased hearts, with diseases encompassing prolapse, stenosis, endocarditis, fibrillation, tachycardia, hypertrophy, mitral calcification, to name a few. The presence of extreme noise present in ultrasound videos was exacerbated to some extent by the lossy compression technique used in storing YouTube videos, which made accurate estimations even more difficult. After fine-tuning the hyperparameters using the aforementioned dataset, we use another dataset to test the robustness of the hyperparameter values. The testing dataset contains 13 videos, of which 10 are healthy and 3 diseased.

It should also be noted here that all the videos were taken without the presence and influence of the authors, meaning that the datasets were from a diverse range of sources, where the subjectivity of the technicians acquiring the datasets made them very challenging to work with, as they had no directions from the authors while obtaining the echocardiograms. This includes the data obtained from the cardiology clinic, as the videos provided by them were of routine patient exams prior to them being approached by the authors. This was intentionally done, in order to illustrate the robustness of the proposed scheme, and the ability to detect the presence of heart diseases effectively from data acquired from any kind of source.

### 3.1. Effects of Varying Values of Parameters

In this section, we observe how changing the values of hyperparameters affects the accuracy of our proposed method. There are seven hyperparameters in our method, namely (a) the number of prongs, (b) the angle of the cone of these prongs, (c) the size of the box on the bottom right corner (in percent w.r.t the size of the frames), (d) the size of the closed boundary within the box (in percent w.r.t the size of the box), the two thresholds being used, (e) the percentage amount of frames permissible to be in the ‘Open’ or ‘Closed’ state while being classified as healthy, and (f) the number of rapid transitions between three consecutive frames that would be tolerated before the heart would be classified as diseased, and finally (g) the step size.

The ideal values chosen for these parameters are respectively, (a) 5 prongs, (b) a cone angle of 40 degrees, (c) a box size in the bottom right corner, 60 percent of the frame size, (d) a closed boundary at least 10% of the dimensions of the box, (e) up to 85% of the frames allowed to be either ‘Open’ or ‘Closed’, (f) the number of allowable rapid transitions should be 5% of the number of frames, and (g) a step size of 0.005. Keeping these parameter values, we tune any one value and measure the accuracy and (in two cases) elapsed time based on these changes. Please note that we have measured elapsed time across only three parameters, as we have found that the other four have no noticeable change in computational time.

#### 3.1.1. Number of Prongs

**Figure 5:**
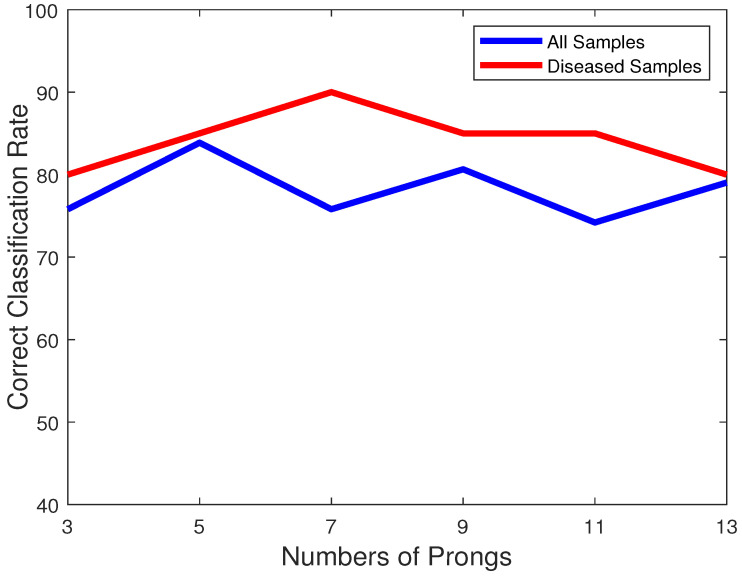
Plot of classification accuracies of all samples (blue) and only diseased samples (red) vs. the number of prongs.

The number of prongs used, will affect how effectively the mitral valve leaflets are being tracked. Too few prongs will give a poor idea of the location of the mitral valve leaflets, and will thus impact its effectiveness. However, too many prongs will make it susceptible to speckle noise present in the images, and erroneously confuse those specks with part of the mitral valve. This trade-off necessitates choosing an optimal number of prongs. It should be noted that since the cone angle is fixed, increasing the number of prongs will make them span the same area with a higher density. Figure 5 plots the variation of accuracies over all the samples (blue) and the diseased samples only (red) with respect to the change of the numbers of prongs. We can observe that for 7 prongs, though the number of diseased hearts are better classified, the overall accuracy is lower. On the other hand, for 5 prongs, the accuracies provide the best trade-off and is thus chosen as the best value. It should also be noted that the number of prongs is odd to ensure the middle prong going through the center of the mitral valve leaflets. This is because although the size of the opening of the leaflets might vary, the middle prong will most easily notice an opening of the leaflets.

#### 3.1.2. Cone Angle of Prongs

**Figure 6:**
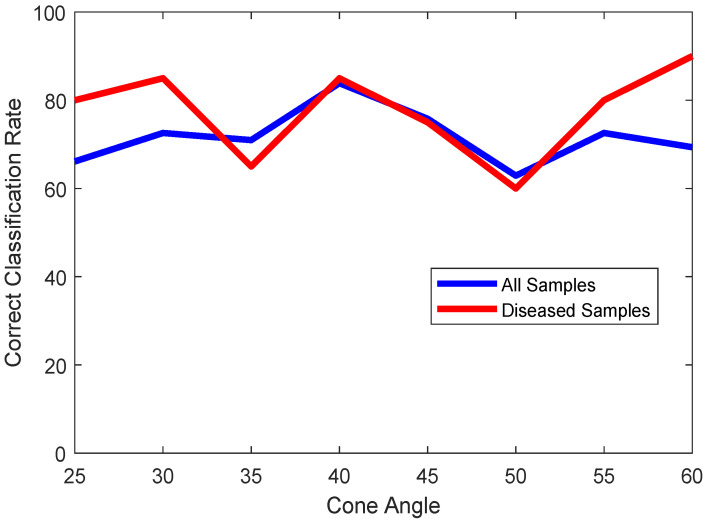
Plot of classification accuracies of all samples (blue) and only diseased samples (red) vs. cone angles of prongs.

The angle of the cone that the prongs encompass is another hyperparameter. An angle too large is going to observe the walls of the left atrium as well, while an angle too small will observe only the center part of the mitral valve leaflets. Thus, a trade-off needs to be studied here. Figure 6 shows the plot of classification accuracies of all samples (blue) and diseased samples (red) across the varying cone angles for the prongs. As we can observe, for a 40-degree cone, we get the best trade-off between correctly classifying healthy and unhealthy hearts.

#### 3.1.3. Size of Box (Accuracy)

In the apical 4 chamber view, the left atrium will usually be on the bottom right corner. Thus, if a box drawn will encompass the bottom right half of the video frames, the left atrium would reside in this box. However, to compensate for the involuntary movements of the heart causing the left atrium to move away from its original location, it would be safer to make the box slightly larger. This size would be another hyperparameter to consider.

**Figure 7:**
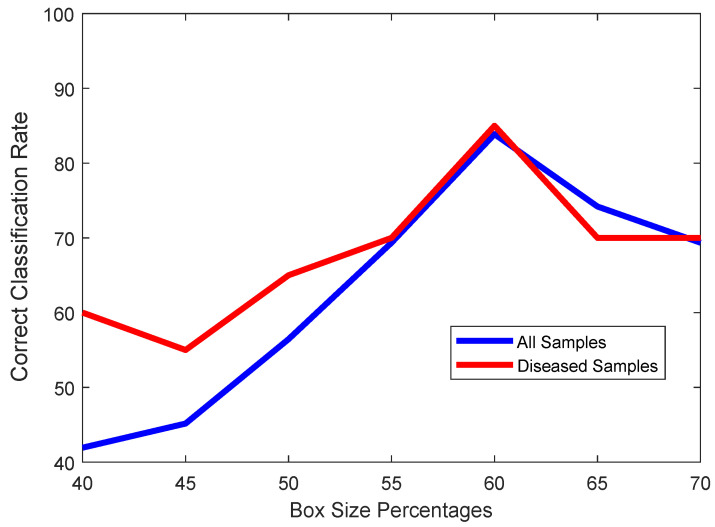
Plot of classification accuracies of all samples (blue) and only diseased samples (red) vs. Percentage of box size regarding the entire frame.

It would be obvious that taking a box too small would cause the left atrium to go outside of this bounding box, and cause the algorithm to either fail to find any closed boundary at all, or it would find some other closed boundary to be the left atrium, which can happen if gaps along the heart muscles occur due to an imperfect alignment of the ultrasound probe. On the other hand, a box that is too large will cause other chambers like the left ventricle to fall within the box, and thus be misclassified as the left atrium. As we can observe in Figure 7, a box with a size of 60% of the frame is the best choice.

#### 3.1.4. Size of Box (Elapsed Time)

**Figure 8:**
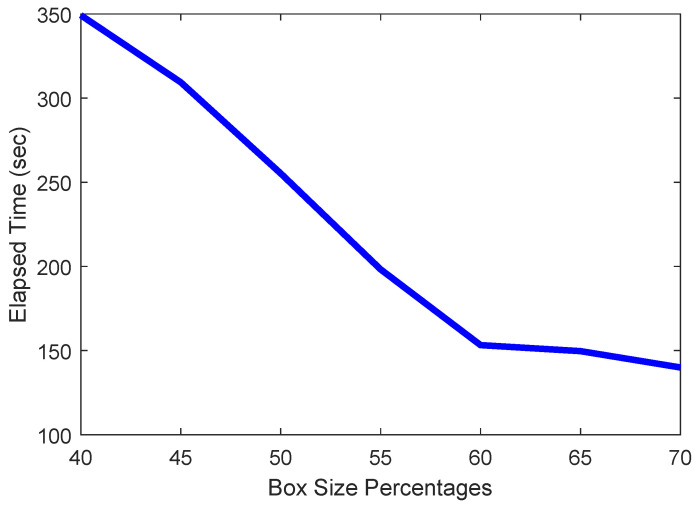
Plot of elapsed time vs. size of box.

When determining the ideal size of the box, what also needs to be observed is the elapsed time. This is because the smaller the box, the harder it is to find a closed boundary within that box, causing the recursive loop in Algorithm 1 to converge more slowly. This will cause the elapsed time to rise significantly, as is attested by the plot in Figure 8. Larger boxes will do to the opposite, finding closed boundaries more easily and thus quickly, but will also sacrifice accuracy significantly.

#### 3.1.5. Contour Size (Accuracy)

**Figure 9:**
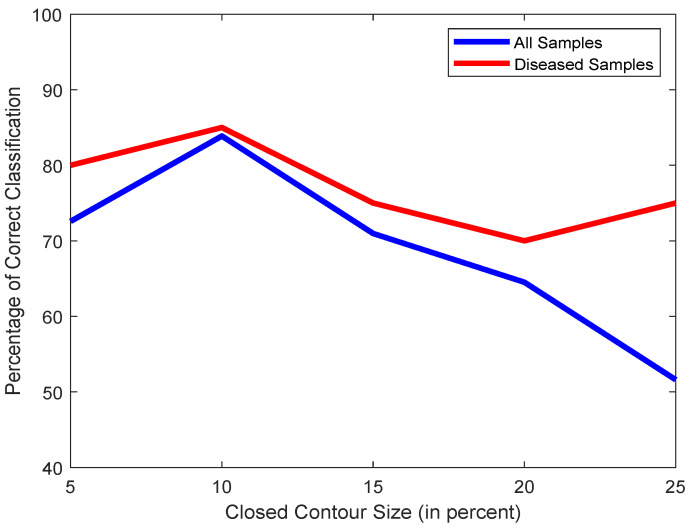
Plot of classification accuracies of all samples (blue) and only diseased samples (red) vs. contour size.

With the box size selected properly, we should carefully choose the correct contour size threshold. A small threshold would make the scheme erroneously consider small objects to be large enough to meet the criteria for being the left atrium, while a threshold too large would result in no contour to be considered large enough to be the left atrium. Figure 9 illustrates the effects of various values as the threshold. We chose 10 as the ideal value.

#### 3.1.6. Contour Size (Elapsed Time)

**Figure 10:**
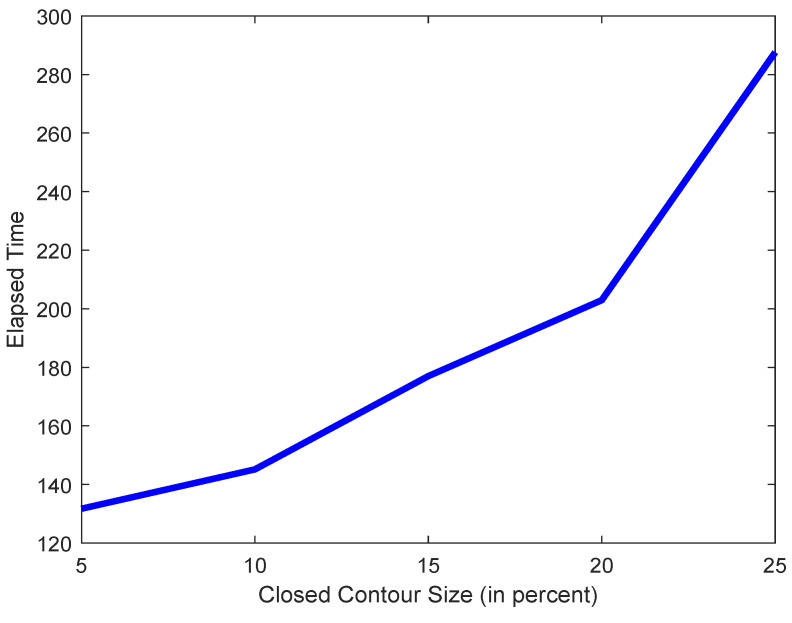
Plot of elapsed time vs. contour size.

As mentioned in Section Figure 9, the smaller the threshold is, the easier, and thus faster it will be for the scheme in Section 2.2 to find a closed boundary. A larger threshold will take the scheme longer to find a contour large enough to meet the threshold. Figure 10 shows this effect, taking longer to converge for higher thresholds.

#### 3.1.7. Open/Close Threshold

**Figure 11:**
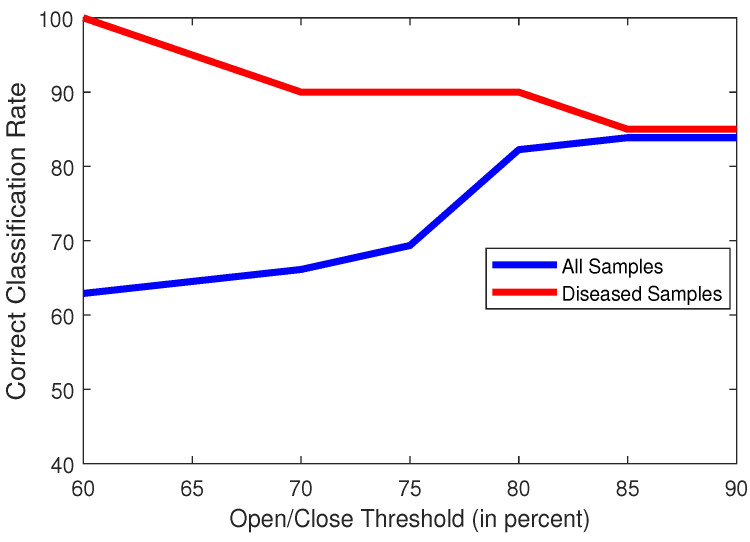
Plot of classification accuracies of all samples (blue) and only diseased samples (red) vs. Open/Close Threshold (in percent of Total No. of Frames).

Another hyperparameter to observe, is the number of frames in a video that can have either an ‘Open’ or ‘Closed’ state, while still considering the heart to be healthy. This is a crucial observation, as the number of frames in the ‘Open’ or ‘Closed’ state will be directly affected by diseases affecting the mitral valve, such as mitral valve stenosis or prolapse. Since we have a dataset where the number of frames can vary greatly, ranging from 16 to 120 frames, it would be difficult to choose a fixed number as the threshold. We have thus chosen to select a percentage of the number of frames as a viable threshold. Figure 11 shows the effect of accuracy across various values of percentage of the total number of frames. Our chosen threshold was 85%.

#### 3.1.8. Transition Threshold

**Figure 12:**
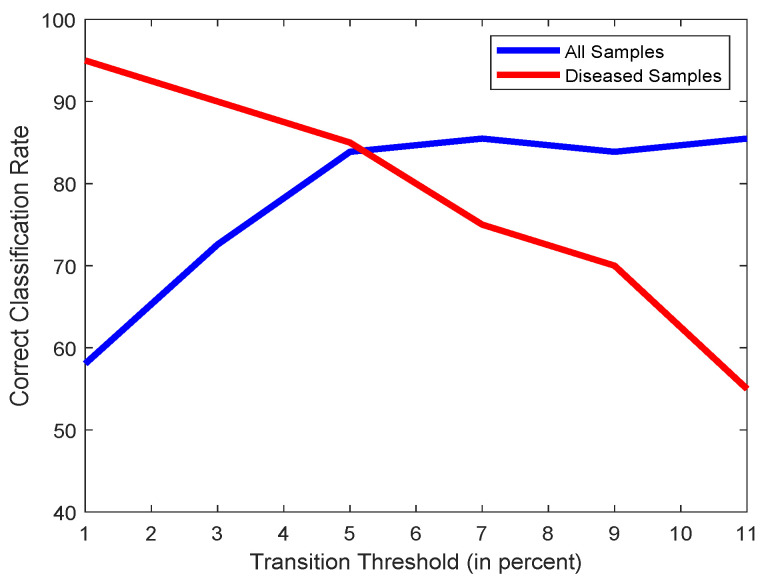
Plot of classification accuracies of all samples (blue) and only diseased samples (red) vs. Open/Close Threshold (in percent of Total No. of Frames).

A healthy heart will usually have a regular mitral valve movement where the transition between ‘Open’ and ‘Closed’ states would likely happen every tens of frames. However, when dealing with diseased hearts that cause the mitral valve leaflets to transition between ‘Open’ and ‘Closed’ states excessively quickly, it would create cases where the leaflets might switch back and forth between ‘Open’ and ‘Closed’ over 3 consecutive frames, necessitating close observation of such cases. However, as such rapid switches might be erroneously caused by observing noise, a threshold needs to be set that will allow a few of these cases to happen in a healthy heart. A threshold too low will be uncompromising and have any misclassified transitions as symptoms of a diseased heart, and a threshold too high will cause an excessively relaxed system that will be unable to detect diseased hearts. Figure 12 shows the effects of the variations of this threshold, and we have thus chosen 5% of the total number of frames to be an ideal threshold of choice.

#### 3.1.9. Step Size (Accuracy)

**Figure 13:**
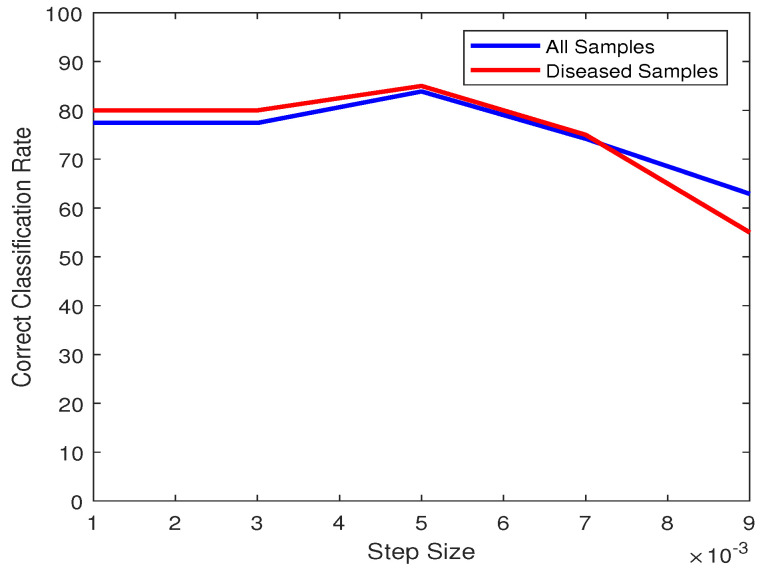
Plot of classification accuracies of all samples (blue) and only diseased samples (red) vs. Step Size.

The last parameter to study is the step size for the recursive loop in Algorithm 1. It should be small enough that the desired threshold value for binarizing is obtained. We can observe in Figure 13 that even though compared to 0.005, one sample gets misclassified for the step size being 0.001 and 0.003, the accuracy is quite stable until 0.005. Increasing the step size beyond this value will cause the accuracy to begin dropping significantly.

#### 3.1.10. Step Size (Elapsed Time)

**Figure 14:**
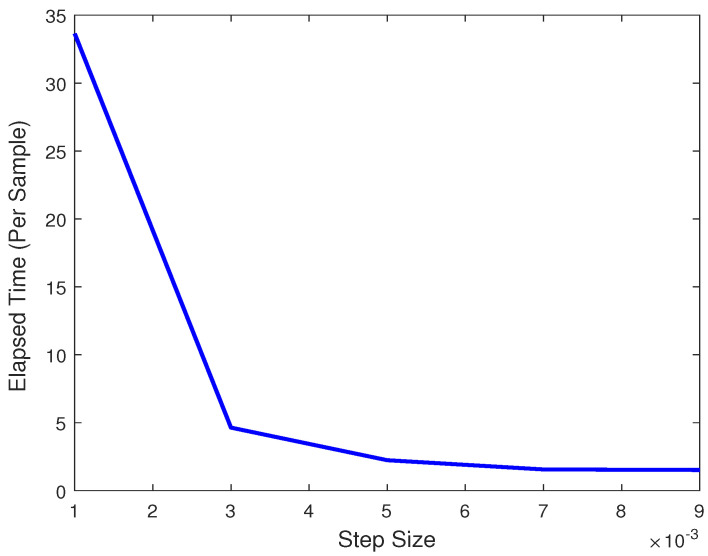
Plot of elapsed times per sample vs. Step Size.

The variation across the step size plays a significant role in the elapsed time per sample. Obviously, a much smaller step size will cause the elapsed time to increase significantly, while a larger step size will result in a lower time. As we can observe in Figure 14, the elapsed time drops significantly when increasing its magnitude, but approaches a stable value beginning from 0.005. As this value is also an ideal choice for best accuracy, 0.005 is the value of choice for the step size.

### 3.2. Computational Complexity

There are a few parts in the algorithm that affect the computational complexity. If we take the number of frames to be *N* and w×h=D, then the binarizing step would have a complexity of O(N×D), and the same would apply for measuring the centroids. For measuring the boundaries, the complexity here across all frames would be O(N). The complexity for Algorithm 1 is a constant since it is not affected by *N* being for one frame only. Next, the calculation of the centroid of the obtained closed boundary would simply be proportional to the size of the frame, so the complexity would be O(D). Assume the number of prongs protruding from the centroid, and the number of points in each prong are denoted by $n_{pr}$ and $n_{pp}$ respectively. Thus, the complexity of calculating the prongs, and their standard deviation would be $O(n_{pr}\;\times \;n_{pp})$, and $O(N\;\times \;n_{pr}\;\times \;n_{pp})$ respectively. Finally, the estimation of frames being open or closed is simply O(N).

### 3.3. Comparison with Other Methods

To test the efficacy of our proposed method, it was compared primarily with two other methods [15,16]. Ref. [16] used graph cuts with a lower rank approximation of a matrix containing the pixel information of the video, to find the faster moving pixels, which would contain the mitral valve as it was presumed that all other parts of the heart would not move as fast. As in the original paper, the frames were cropped to contain only the left chambers, the same was done with our data when applying this method. To apply this scheme, we used the graph cut method in [25,26,27,28]. This method will be referred to as LRRGC (Low-Rank Representation Graph Cuts). As the scheme did not classify as to whether the valve was open or not, a manual feature was added, which would observe the span of the area tracked by the algorithm, and see how it is spread out. A valve that is horizontal is likely to be closed, while being vertical would be open.

The method in [15] does not work in an unsupervised manner, instead it relies on one supervised part. The coordinates of the initial point are hand-picked by the user, which is the point where the anterior mitral leaflet connects with the wall of the atrium. The anterior mitral leaflet is the larger of the two leaflets that make up the mitral valve. After that, estimation of the leaflet is unsupervised. This method will be referred to as the VM-Mode.

**Figure 15:**
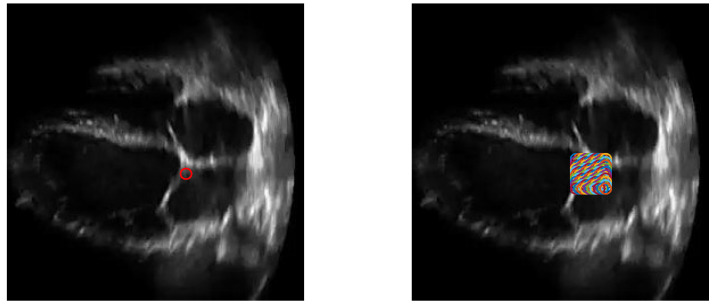
An ultrasound image with one initialization point (**left**) and 100 points (**right**).

One thing to note is that the method in [15] worked with the parasternal long axis view, where the mitral leaflet’s movements are horizontal. However, our proposed method works with the apical 4 chamber view, where the mitral valve’s movements are vertical. To accomodate this, the apical 4 chamber videos were rotated to fit the perspective of the views that [15] is accustomed to. As a form of compensation, in comparing the performance of our proposed method with [15], the comparative method has been given a major advantage. As the initial point has to be selected carefully, we have designed a scheme where the method will not only take the given initial point, but it will consider the points in a box around the selected point. The size of this box will be 10% of the length of the frame across both dimensions, and will have 10 equidistant points on each dimension. In other words, instead of using only one point, the method will use 100 points from a box of size 0.1∗w×0.1∗h around the given point, and so 100 iterations of the method will be taken. Among these, the iteration with the highest accuracy will be chosen as the ‘true’ initialization point. Figure 15 shows the difference between taking 1 point only, and taking a box of points around the given point.

To further show the effectiveness of our application of our binarization scheme, including the recursive loop shown in Algorithm 1, we have applied two other methods to our scheme. The work in [29] uses an adaptive Otsu thresholding and K-means clustering to binarize the image. We have implemented this method instead of the binarizing scheme that we have developed, and recorded the results. We have referred to this implementation as KMOtsu (K-Means Otsu).

On the other hand, Ref. [30] uses a super-resolution method to have a higher resolution version of the obtained images, and uses the Speckle Reducing Anisotropic Diffusion [31] method to reduce speckle noise. We have referred to this method as BUSRD (Breast Ultrasound Super Resolution and Despeckle). This is used in our scheme to obtain upsampled, and despeckled images prior to binarizing, and then fed it through our algorithm. The code for the SRAD algorithm was obtained from [32].

Table 1 shows the comparison among the four methods discussed. Both methods were implemented in conjunction with Section 2.5 in order to obtain performance results. The figures of merit to use were (i) the number of videos correctly classified as healthy or diseased, and (ii) the time taken to obtain these results. The times per sample were obtained by simply taking the average time to run the algorithm for all 62 samples. The method with better results for each parameter is highlighted in bold. As we can observe, our novel method was performing better than the VM-Mode [15], even though the latter is supervised and is given an advantage. In order to provide an idea of the severity of the advantage given to [15], the performance of the VM-Mode without the advantage is also shown. Clearly, the improvement with the advantage is minimal. Furthermore, the number of classified diseased hearts did not improve at all.

On the other hand, for LRRGC, the issue was that due to several the hearts with significant involuntary movement, parts like the interatrial septum were also moving too fast to be considered to be part of the background. This led to the scheme erroneously segmenting these parts as belonging to the mitral valve. Because of this, even if the valve was closed, it would be observed as open. As this caused a significant impact in the misclassification of the frames, it also caused most of the frames to be estimated as being in the ‘closed’ state, causing a lower accuracy in frames, and it also meant that many healthy hearts were misclassified as diseased. Consequently, it also caused a higher rate of videos that were actually diseased, to be coincidentally correctly classified. However, due to the complexity of the scheme, as well as dealing with extremely large arrays containing pixel information of an entire video, this came at great computation cost, causing much higher runtimes.

For KMOtsu, the improved Otsu’s thresholding alone is unable to effectively observe the thin mitral valve tissues in the binarized image. On top of that, the interatrial septum will also be difficult to observe, given its extreme thinness. This will cause the method to be less robust when finding a closed contour around the left atrium, as this is incompatible with the scheme introduced in Algorithm 1. As a result, often this method will often make it impossible to locate the mitral valve in the images.

In the case of BUSRD, while it is indeed effective in reducing speckle noise, it also runs the risk of removing at least parts of the interatrial septum and mitral valve tissues. This will cause the scheme to face similar obstacles like the KMOtsu method, since it might make it impossible to obtain a closed boundary around the left atrium, as well as make it difficult to observe tissues as thin as the mitral valve leaflets. Please note that the thinness of the mitral valve leaflets is especially concerning in our datasets, because in these videos, there was no distinct priority on the technicians’ perspective in acquiring videos where the mitral valve leaflets can be observed as clearly as possible.

We have also added the precision, recall and f1 square values of the methods. Precision is the ratio of true positives (correctly classifying healthy hearts) and the sum of true positives with false positives (misclassifying diseased hearts as healthy). On the other hand, recall is the ratio of true positives, and the sum of true positives with false negatives(misclassifying healthy hearts as diseased). The f1 score is basically $2\frac{precision\times recall}{\left(precision+recall\right)}$. We can see that the proposed method outperforms all the other methods, except for in Recall, which is understandable as the VM-Mode was unable to classify any heart as diseased, giving the number of false negatives to be zero, and thus making the ratio equal to 1.

As mentioned in the beginning of this section, after finding the optimal values for the hyperparameters, and testing across the dataset of 62 videos, we include another testing set of videos where the hyperpameters’ values were unchanged. Table 2 shows the numerical results from the second dataset. KMOtsu erroneously classified a majority of the videos since it was unable to locate the mitral valve, thus creating prongs in incorrect locations. LRRGC, on the other hand, erroneously classified all the videos as diseased, causing the number of true positives to be zero, thus causing the Precision and F1 Score to be infinite. The supervised VM-Mode had a slight advantage in that it classified one more video correctly, compared to the proposed method. However, it can be easily observed that the proposed method, while being unsupervised, is highly consistent over the two datasets. There is also another key point to note here, which is that when providing the advantage with VM-Mode, it actually misclassifies all the diseased samples. This is because as we are taking a 10x10 window around the seed point, none of those points might have exactly the same values as the original seed point, but rather be a very close value. The fact that this slight difference can cause a misclassification, can further attest to the robustness of the unsupervised proposed method.

## 4. Conclusions

A novel unsupervised method was created for estimating the movement of the mitral valve, as well as detecting the state of health of the heart in echocardiogram videos. A scheme was developed where estimation was made much faster by binarizing the frames of the videos with Otsu’s thresholding. The frames had background subtraction to isolate the mitral valve in motion, and centroids were taken of the frames with background subtraction, as the farthest centroids pointed to valves that were fully in the open and closed states. These were used to obtain a closed boundary around the left atrium, from which protruding prongs were taken to measure the exact positions of the mitral valve leaflets at any given frame. With successful estimation of the positions of the leaflets, estimations were made as to the ‘open/closed’ states of the valve, which gave an insight into its movement patterns. This was then used to determine the health of the heart with respect to diseases that may affect movement of the mitral valve. Despite facing issues inherent in ultrasound videos, such as strong noise, and involuntary movement of the heart making a fully unsupervised estimation scheme difficult, our proposed method was shown to be effective through numerical testing, even while compared to a supervised method, as well as an unsupervised method. Potential avenues of further improvement, can include schemes for automatically determining the cone angle of the prongs, since depending on the width of the mitral valve opening, the results might improve based on automated fine-tuning of the angles. Another scope for improvement would be finding an automated way to realign videos where the heart may be tilted at a slight angle, as this can occur due to the subjective preference of the technician obtaining the echocardiogram. Please note that this improvement can also be done with tilting the prongs at the corresponding angle as well. We should emphasize that our preferences for improvement would focus on further automation of our scheme, as unsupervised heart disease detection is a body of work that is underdeveloped, but has great potential.

## Figures and Tables

**Table 1:** Performance Comparison between Proposed method, VM-Mode [15], LRRGC [16], KMOtsu [29] and BUSRD [30] for the first dataset (bold indicates best value).

Test Parameters	Proposed Method	VM—Mode (with Advantage)	VM—Mode (without Advantage)	LRRGC	KMOtsu	BUSRD
% of Correct Classification (All)	**83.87**	64.71	63.27	32.26	27.42	61.29
% of Correct Classification (Diseased)	85.00	0	0	**95**	70.00	65.00
Mean Time(s) per Sample	2.11	2.63	2.81	39.58	**1.99**	137.21
Mean Time(s) per Frame	**0.03**	0.04	0.04	0.61	0.27	1.92
Precision	**0.92**	0.65	0.64	0.50	0.75	0.78
Recall	0.83	**1.00**	**1.00**	0.02	0.09	0.61
F1 Score	**0.88**	0.79	0.78	0.05	0.16	0.68

**Table 2:** Performance Comparison between Proposed method, VM-Mode [15], LRRGC [16], KMOtsu [29] and BUSRD [30] for the second dataset (bold indicates best value).

Test Parameters	Proposed Method	VM—Mode (with Advantage)	VM—Mode (without Advantage)	LRRGC	KMOtsu	BUSRD
% of Correct Classification (All)	84.62	76.92	**92.31**	23.08	23.08	61.54
% of Correct Classification (Diseased)	**100**	0.00	66.67	**100**	66.67	66.67
Mean Time(s) per Sample	**3.36**	3.43	3.88	45.98	13.34	105.37
Mean Time(s) per Frame	**0.04**	**0.04**	0.05	0.42	0.17	1.34
Precision	**1.00**	0.77	0.91	Inf	1.00	0.86
Recall	0.80	1.00	**1.00**	0.00	0.11	0.60
F1 Score	0.89	0.87	**0.95**	Inf	0.20	0.71
